# Sauna bathing is associated with reduced cardiovascular mortality and improves risk prediction in men and women: a prospective cohort study

**DOI:** 10.1186/s12916-018-1198-0

**Published:** 2018-11-29

**Authors:** Tanjaniina Laukkanen, Setor K. Kunutsor, Hassan Khan, Peter Willeit, Francesco Zaccardi, Jari A. Laukkanen

**Affiliations:** 1 Institute of Public Health and Clinical Nutrition, University of Eastern Finland, P.O. Box 1627, FIN-70211 Kuopio, Finland; 2 Central Finland Health Care District, Jyväskylä, Finland; 3 National Institute for Health Research Bristol Biomedical Research Centre, University Hospitals Bristol NHS Foundation Trust and University of Bristol, Bristol, UK; 4 Translational Health Sciences, Bristol Medical School, Musculoskeletal Research Unit, University of Bristol, Learning & Research Building (Level 1), Southmead Hospital, Bristol, UK; 5 Division of Cardiology, Department of Medicine, Emory University, Atlanta, GA USA; 6 Department of Public Health and Primary Care, University of Cambridge, Cambridge, UK; 7 Department of Neurology, Medical University Innsbruck, Innsbruck, Austria; 8 Diabetes Research Centre, Leicester General Hospital, University of Leicester, Leicester, UK; 9 Faculty of Sport and Health Sciences, University of Jyväskylä, Jyväskylä, Finland

**Keywords:** Sauna bathing, Prevention, Cardiovascular disease, Gender, Risk prediction

## Abstract

**Background:**

Previous evidence indicates that sauna bathing is related to a reduced risk of fatal cardiovascular disease (CVD) events in men. The aim of this study was to investigate the relationship between sauna habits and CVD mortality in men and women, and whether adding information on sauna habits to conventional cardiovascular risk factors is associated with improvement in prediction of CVD mortality risk.

**Methods:**

Sauna bathing habits were assessed at baseline in a sample of 1688 participants (mean age 63; range 53–74 years), of whom 51.4% were women. Multivariable-adjusted hazard ratios (HRs) were calculated to investigate the relationships of frequency and duration of sauna use with CVD mortality.

**Results:**

A total of 181 fatal CVD events occurred during a median follow-up of 15.0 years (interquartile range, 14.1–15.9). The risk of CVD mortality decreased linearly with increasing sauna sessions per week with no threshold effect. In age- and sex-adjusted analysis, compared with participants who had one sauna bathing session per week, HRs (95% CIs) for CVD mortality were 0.71 (0.52 to 0.98) and 0.30 (0.14 to 0.64) for participants with two to three and four to seven sauna sessions per week, respectively. After adjustment for established CVD risk factors, potential confounders including physical activity, socioeconomic status, and incident coronary heart disease, the corresponding HRs (95% CIs) were 0.75 (0.52 to 1.08) and 0.23 (0.08 to 0.65), respectively. The duration of sauna use (minutes per week) was inversely associated with CVD mortality in a continuous manner. Addition of information on sauna bathing frequency to a CVD mortality risk prediction model containing established risk factors was associated with a C-index change (0.0091; *P* = 0.010), difference in − 2 log likelihood (*P* = 0.019), and categorical net reclassification improvement (4.14%; *P* = 0.004).

**Conclusions:**

Higher frequency and duration of sauna bathing are each strongly, inversely, and independently associated with fatal CVD events in middle-aged to elderly males and females. The frequency of sauna bathing improves the prediction of the long-term risk for CVD mortality.

**Electronic supplementary material:**

The online version of this article (10.1186/s12916-018-1198-0) contains supplementary material, which is available to authorized users.

## Background

Sauna bathing, a form of passive heat therapy, is a traditional activity in Finland and widely used for relaxation purposes and is becoming increasingly common in many other countries [1–4]. Emerging evidence suggests that sauna bathing is linked with several health benefits, including a reduction in the risk of high blood pressure or hypertension [5, 6], stroke [7], neurocognitive diseases [8], and pulmonary diseases [9–11]. Sauna bathing has also been used in treating musculoskeletal pain [12, 13] as well as chronic headache [14]. The beneficial effects of sauna bathing on these adverse events have been linked to its positive impact on circulatory and cardiovascular function. It has been suggested that regular heat therapy may improve cardiovascular function via improved endothelium-dependent dilatation, reduced arterial stiffness, modulation of the autonomic nervous system, and lowering of blood pressure [6, 15–18].

We have shown that having frequent sauna baths is strongly associated with a reduced risk of fatal cardiovascular outcomes and all-cause mortality in a general population sample of middle-aged men [19]. To our knowledge, this is the only available study [19] on the prospective association between sauna habits and the risk of mortality outcomes. It is therefore unknown whether the additional cardiovascular benefits of frequent sauna bathing are also applicable to women and older individuals. In addition, there is no data on the associations of both weekly frequency and duration of sauna bathing with a risk of cardiovascular disease (CVD) in populations including men and women. Furthermore, given the strong independent association between sauna bathing and the risk of CVD, there is a possibility that adding information on sauna bathing habits to current CVD risk prediction algorithms might be associated with improvements in the ability to predict CVD risk. The potential utility of sauna bathing for CVD risk assessment has not yet been evaluated, and therefore, this warrants investigation. In this context, we aimed to evaluate the relationship between sauna bathing habits (both frequency and duration) and the risk of CVD mortality in a large population-based cohort of middle-aged to elderly men and women. We also investigated the extent to which information on sauna habits could improve the prediction of CVD mortality in our study population using measures of risk discrimination and reclassification.

## Methods

### Study design

**Fig. 1:**
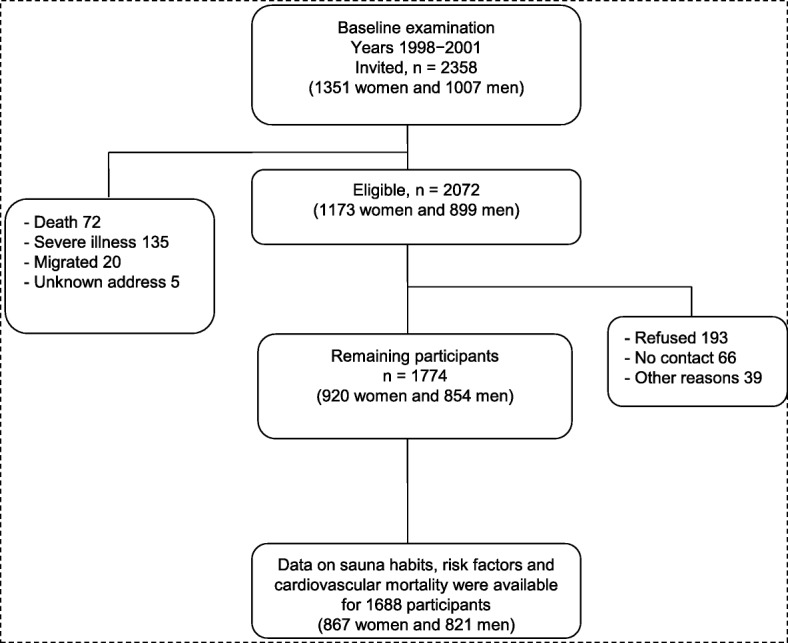
Flowchart of the prospective study setting included in the analyses on sauna bathing and fatal cardiovascular outcomes

We employed the Kuopio Ischaemic Heart Disease (KIHD) Study, which is a population-based prospective cohort study designed to investigate sauna bathing habits and other risk factors for CVD [19, 20]. The KIHD Study was initially based on a cohort of men aged 42–61 years who were living in Kuopio and the surrounding rural communities in the east of Finland. In the 11-year follow-up visit of the first cohort, women were invited to join this study. In this cohort which is being utilized for this analysis, participants (*n* = 2358) comprised a randomly selected sample of 1351 women and 1007 men aged 53.4 to 73.8 years. Of 2072 eligible participants, 1774 participated in the current prospective sauna study. We excluded 31 participants without information on assessment of sauna bathing habits at baseline examination. Of the remaining participants, complete data on sauna bathing, clinical characteristics, biomarkers, and fatal CVD outcomes were available for 1688 participants (867 women and 821 men) (Fig. 1). All baseline examinations were carried out between March 1998 and December 2001. This study was performed following the STROBE (STrengthening the Reporting of OBservational studies in Epidemiology) guidelines for reporting observational studies in epidemiology (Additional file 1: Appendix) [21].

### Assessment of sauna bathing

In a traditional Finnish sauna, there is dry air with a relative humidity of about 10–20%. It is possible to increase the humidity temporarily by throwing water on the hot rocks of the sauna heater, although it usually remains below 20%. The recommended temperature for sauna is from 80 to 100 °C at the level of the bather’s head, but the temperature is much lower at the floor level (about 30 °C) which keeps the ventilation of the sauna room efficient and sauna condition comfortable for sauna bathers [5]. The duration of stay in the sauna room depends on the comfort and temperature of the sauna bather, but it usually ranges from 5 to 20 min, although the sessions could be longer depending on the individual [22]. In the current study, sauna bathing was assessed at baseline by a self-administrated questionnaire based on weekly sauna sessions, duration, and temperature in the sauna room [19]. The assessment represents a typical sauna use during the week, and the temperature in the sauna room was measured using a thermometer. The questionnaires were checked by an experienced nurse at the time of baseline examination.

### Assessment of risk factors and baseline characteristics

Risk factors and all other characteristics were assessed during the same visit at study entry. Baseline demographics and socioeconomic and living condition characteristics were assessed among the study participants. A participant who had ever smoked on a regular basis was defined as a smoker. The use of medications, baseline diseases, the level of physical activity, and socioeconomic status (SES) were assessed by self-administered questionnaires [23]. The total and energy expenditure of physical activity was assessed from a validated 12-month leisure time physical activity questionnaire [24, 25]. This detailed quantitative questionnaire deals with the most common leisure time physical activities of middle-aged Finnish men. For the type of physical activity performed, participants were asked to document the frequency (number of sessions per month), average duration (hours and minutes per session), and intensity [26]. Energy expenditure was measured for each physical activity by multiplying the metabolic index of activity (in metabolic equivalent × hour/week) by body weight in kilograms. The diagnosis of chronic diseases and medication was assessed during a medical examination by a doctor. Alcohol consumption was assessed using the Nordic Alcohol Consumption Inventory [27]. Dietary energy intake was assessed using 4-day food recording (Nutricia); instructions were given, and completed food records were checked by a nutritionist. Resting blood pressure was measured between 8 and 10 a.m. with a random-zero sphygmomanometer. Participants were instructed to fast overnight, abstain from alcohol consumption for at least 3 days, and to keep away from smoking for at least 12 h prior to blood specimen collection. The cholesterol contents of serum lipoprotein fractions and triglycerides were measured enzymatically (Boehringer Mannheim, Mannheim, Germany). Serum high-density lipoprotein and its subfractions were separated from fresh serum samples using ultracentrifugation and precipitation. Body mass index (BMI) was computed as the ratio of weight in kilograms to the square of height in meters.

### Ascertainment of outcomes

All CVD deaths that occurred by the end of 2015 were checked against the hospital documents, health center wards and death certificates, and medico-legal reports [28]. There were no losses to follow-up. All participants (just like every individual in Finland) have personal identity codes which are annually matched through computerized linkage with registries for hospitalizations, discharges, and deaths. Annual follow-up for outcomes is also done automatically using the personal identifiers. Registries are also regularly linked with the Central Population Register to ensure that the personal identity codes are correct. Cardiovascular disease deaths were coded using the Tenth International Classification of Diseases codes. Data on incident coronary heart disease (CHD) events from the beginning of the study were based on the national discharge registers [23]. The documents related to the death were cross-checked in detail by two physicians.

### Statistical analysis

Differences in baseline characteristics were examined using the analysis of variance, the independent samples *t* test, and the chi-squared test. Descriptive data are presented as means (standard deviation, SD) and percentages. Hazard ratios (HRs) with 95% confidence intervals (CIs) for CVD mortality were calculated using Cox proportional hazard models after confirming the assumptions of the proportionality of hazards using Schoenfeld residuals [29]. Subjects were classified into groups on the basis of frequency of sauna bathing (1, 2–3, and 4–7 times per week) and the total weekly duration of a sauna bathing (≤ 15, 16–45, > 45 min/week) to maintain consistency with previous reports [6, 8, 19, 30]. In a subsidiary analysis, we categorized frequency of sauna bathing into 0–1, 2–3, and 4–7 times per week, including participants who did not use sauna at all (*n* = 43). Hazard ratios of the associations of frequency and duration of sauna bathing with CVD mortality were progressively adjusted for age and gender (model 1); BMI, smoking, systolic blood pressure (SBP), serum low-density lipoprotein cholesterol (LDL-C), alcohol consumption, previous myocardial infarction, and type 2 diabetes (model 2); total duration of physical activity per week and SES (model 3); and incident CHD events as a time-varying covariate (model 4), as it is a known factor in the pathway for development of CVD mortality. Covariates were selected on the basis of their previously established roles as well-defined predictive or confounding factors, evidence from previous research, or their potential as confounders based on known associations with cardiovascular outcomes and observed associations with sauna exposure using the available data [31]. The cumulative survival from CVDs according to the frequency and duration of sauna bathing was calculated using the Kaplan-Meier method. We explored the shape of the relationship between the frequency of sauna bathing and CVD mortality, using restricted cubic spline with knots at the 5th, 35th, 65th, and 95th percentiles of the distribution of sauna frequency in a multivariate-adjusted model. We also characterized the shape of the association between duration of sauna bathing and CVD mortality risk by calculating HRs within the quartiles of the duration of sauna bathing and plotted them against mean sauna bathing duration within each quartile using floating absolute risks. We performed subgroup analyses using interaction tests to assess statistical evidence of any differences in HRs across levels/categories of pre-specified clinically relevant characteristics such as age at survey, gender, BMI, SBP, total cholesterol, LDL-C, high-density lipoprotein cholesterol (HDL-C), total duration of physical activity per week, energy expenditure of physical activity, history of diabetes mellitus, smoking status, history of hypertension, and prevalent CHD. To minimize biases due to reverse causation, sensitivity analysis involved excluding the first 5 years of follow-up.

To assess whether adding information on the frequency of sauna bathing (main exposure) to conventional cardiovascular risk factors would result in an improvement in the prediction of CVD mortality risk, we calculated measures of discrimination for censored time-to-event data (Harrell’s C-index [32]) and reclassification [33, 34]. To investigate the change in C-index on the addition of frequency of sauna bathing, two CVD mortality risk prediction models were fitted: one model based on traditional risk factors (i.e., age, sex, SBP, history of diabetes, total cholesterol, HDL-C, and smoking) and the second model with these risk factors plus frequency of sauna bathing. Reclassification analysis was restricted to the first 10 years of follow-up and was assessed using the net reclassification improvement (NRI) [33] and integrated discrimination improvement (IDI) [33]. Reclassification analysis was based on predicted 10-year CVD mortality risk categories of low (< 1%), intermediate (1 to < 5%), and high (≥ 5%) risk as previously reported [35]. Given that Harrell’s C-index can be very insensitive in detecting differences in risk prediction analyses [36, 37], to avoid discarding potential biomarkers that can be used in risk prediction, it has been recommended to also use sensitive risk discrimination methods such as the − 2 log likelihood test [36, 37]. Therefore, in addition to Harrel’s C-index, we tested for differences in the − 2 log likelihood of prediction models with and without the inclusion of frequency of sauna bathing. A *P* value < 0.05 was considered statistically significant. Statistical analyses were performed using Stata version 12 (Stata Corp, College Station, TX).

## Results

### Baseline characteristics

A summary of the baseline characteristics of overall study participants and according to the group of weekly frequency of sauna bathing is shown in Table 1. There were 867 (51.4%) female and 821 (48.6%) male participants. The mean (SD) age, BMI, and waist-to-hip ratio were 63 years (7), 27.9 kg/m^2^ (4.4), and 0.91 (0.09), respectively. The median (interquartile range, IQR) frequency and duration of sauna bathing were two (one to three) sessions and 30 min (15–45) per week, respectively. The mean (SD) temperature of the sauna bath was 75.9°C (9.9). The average temperature of sauna room was slightly lower (74.8 °C) among participants who had four to seven sauna bathing sessions per week compared to those with only 1 sauna bathing session per week (77.4 °C). Participants with a frequency of sauna bathing of four to seven sessions per week had higher BMI and alcohol and energy intake, compared to those with 1 sauna session per week. When comparing men to women in terms of median frequency and duration of sauna bathing, the median (IQR) values were two (two to three) vs. two (one to two) sessions per week and 30 (20–45) vs. 20 min (13–30) per week, respectively; the mean (SD) temperature of the sauna bath was 77.1 (9.0) vs. 74.7 °C (10.5) for men and women, respectively.

**Table 1:** Baseline characteristics of overall study participants and according to frequency of sauna bathing

Characteristics		Frequency of sauna bathing (times per week)			
	Overall (*N* = 1688)	1 (*n* = 455)	2–3 (*n* = 1028)	4–7 (*n* = 205)	*P* value for heterogeneity
	Mean (SD) or n (%) or median (IQR)	Mean (SD) or n (%) or median (IQR)	Mean (SD) or n (%) or median (IQR)	Mean (SD) or n (%) or median (IQR)	
Sauna use					
Temperature, °C	75.9 (9.9)	77.4 (9.4)	75.4 (9.7)	74.8 (10.9)	< 0.001
Duration, minutes/sauna session, median (IQR)	13 (10–15)	10 (10–15)	15 (10–20)	13 (10–15)	0.073
Duration, minutes/week, median (IQR)	30 (15–40)	10 (10–15)	30 (20–40)	60 (40–90)	< 0.001
Demographics					
Age, years	63 (7)	64 (7)	63 (6)	60 (6)	< 0.001
Male, *n* (%)	821 (48.6)	177 (38.9)	512 (49.8)	132 (64.4)	< 0.001
Body mass index, kg/m^2^	27.9 (4.4)	27.4 (4.4)	28.1 (4.4)	28.2 (4.6)	0.013
Systolic blood pressure, mmHg	136 (17)	137 (18)	136 (17)	135 (17)	0.356
Diastolic blood pressure, mmHg	81 (9)	81 (9)	81 (9)	82 (10)	0.259
Alcohol consumption, g/week, median (IQR)	12.20 (1.00–53.68)	8.75 (0.32–45.11)	12.53 (1.29–53.34)	24.00 (3.20–76.60)	0.004
Smokers, *n* (%)	221 (13.1)	70 (15.4)	132 (12.8)	19 (9.3)	0.091
Smoking, pack years*	3.05 (10.3)	3.7 (11.4)	2.9 (10.1)	2.1 (8.3)	0.174
Total physical activity per week, h, median (IQR)^†^	7.94 (4.60–13.21)	7.31 (4.16–12.05)	8.21 (4.80–13.21)	8.48 (4.78–14.76)	0.029
Physical activity, MET h/year, median (IQR)^†^	1817 (1077–2992)	1625 (948–2703)	1874 (1109–3055)	2012 (1256–3296)	< 0.001
Energy expenditure of physical activity, kcal/day, median (IQR)^†^	383 (224–598)	325 (186–512)	399 (237–610)	449 (293–723)	< 0.001
Mean intensity of physical activity, METs^†^	4.58 (1.02)	4.43 (1.02)	4.61 (1.02)	4.76 (1.03)	< 0.001
Energy intake, kJ/day	7612 (2397)	7146 (2263)	7688 (2393)	8250 (2524)	< 0.001
Previous myocardial infarction, *n* (%)	118 (7.0)	34 (7.5)	70 (6.8)	14 (6.8)	0.895
History of coronary heart disease, *n* (%)	474 (28.1)	128 (28.1)	289 (28.1)	57 (27.8)	0.996
Type 2 diabetes, *n* (%)	138 (8.2)	45 (9.9)	80 (7.8)	13 (6.3)	0.233
Hypertension, *n* (%)	702 (41.6)	196 (43.1)	421 (41.0)	85 (41.5)	0.746
Serum LDL cholesterol, mmol/L	3.59 (0.93)	3.56 (0.95)	3.61 (0.93)	3.59 (0.87)	0.598
Serum HDL cholesterol, mmol/L	1.25 (0.31)	1.26 (0.33)	1.24 (0.30)	1.26 (0.33)	0.356
Fasting blood glucose, mmol/L	5.1 (1.2)	5.1 (1.3)	5.1 (1.2)	5.1 (1.2)	0.985
Socioeconomic and living condition characteristics					
Socioeconomic status, unit^‡^	10.9 (4.7)	10.3 (4.7)	11.3 (4.7)	10.3 (4.5)	< 0.001
Annual income (1998–2001), €	16,144 (11,715)	16,970 (10,474)	15,337 (10,703)	18,377 (17,403)	0.001
Academic degree (college or university), *n* (%)	93 (5.5)	47 (10.3)	40 (3.9)	6 (2.9)	< 0.001
Daily working time (duration), h	8.1 (1.8)	7.9 (1.7)	8.1 (1.7)	8.7 (2.4)	< 0.001
Physical strain of work, unit	2.40 (0.88)	2.35 (0.89)	2.42 (0.87)	2.43 (0.87)	0.402
Mental strain at work, unit	2.47 (0.69)	2.47 (0.72)	2.46 (0.68)	2.50 (0.68)	0.748
Type of residence, *n* (%)					< 0.001
Family house	825 (48.9)	106 (23.4)	574 (55.8)	145 (70.7)	
Attached house	219 (13.0)	75 (16.5)	123 (12.0)	21 (10.2)	
Apartment house	643 (38.1)	273 (60.1)	331 (32.2)	39 (19.0)	
Summer cottage (own available), *n* (%)	804 (47.8)	190 (41.9)	512 (50.1)	102 (49.8)	0.013

Complete baseline information was available on 1688 individuals

*IQR* interquartile range, *SD* standard deviation, *LDL* low-density lipoprotein, *HDL* high-density lipoprotein

*Pack-years denotes the lifelong exposure to smoking which was estimated as the product of years smoked and the number of tobacco products smoked daily at the time of examination

^†^Physical activity was computed by multiplying the duration and intensity of each physical activity by body weight. Physical activity was assessed using the 12-month physical activity questionnaire

^‡^Socio-economic status is a summary index that combines measures of income, education, occupation, occupational prestige, material standard of living, and housing conditions, all of which were assessed with self-reported questionnaires

### Sauna bathing and fatal cardiovascular events

**Fig. 2:**
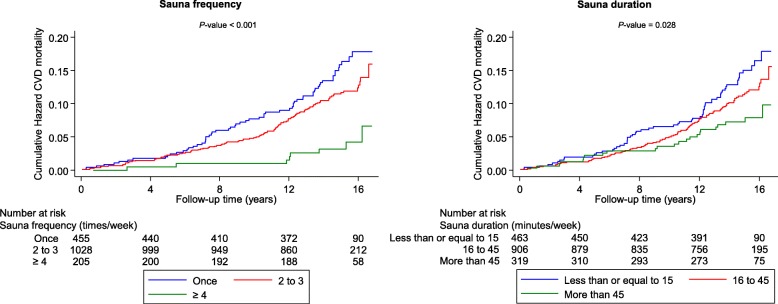
Cumulative Kaplan-Meier curves for cardiovascular mortality according to the frequency and duration of sauna bathing per week

**Fig. 3:**
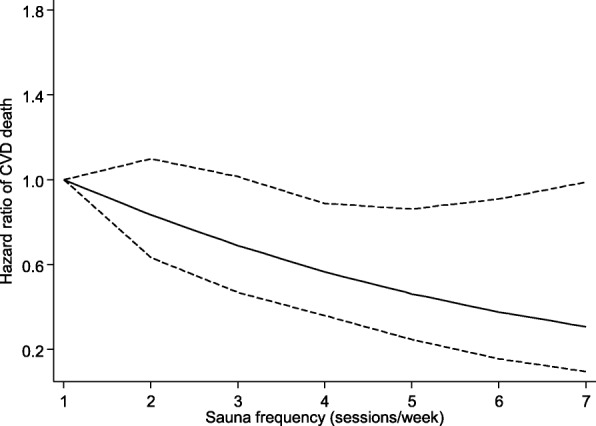
Restricted cubic spline model of the hazard ratios of cardiovascular mortality with the frequency of sauna bathing. Restricted cubic spline functions were analyzed with knots located at 5th, 35th, 65th, and 95th percentiles of sauna bathing frequency distribution, with the reference category set at one session/week; adjusted for age, gender, body mass index, smoking, systolic blood pressure, serum low-density lipoprotein cholesterol, alcohol consumption, previous myocardial infarction, and type 2 diabetes. The dashed lines represent the 95% confidence intervals

During a median (interquartile range) follow-up of 15.0 years (14.1–15.9) (23,601 person-years at risk), a total of 181 CVD deaths occurred. Cardiovascular mortality rates per 1000 person-years across the three frequency groups of sauna bathing (one, two to three, and four to seven times per week) were 10.1 (95% CI 7.9 to 12.9), 7.6 (6.3 to 9.2), and 2.7 (1.3 to 5.4), respectively. According to the frequency of sauna bathing, cumulative hazard curves demonstrated the lowest risk of CVD mortality among participants who had four to seven sauna sessions per week compared to other groups (*P* < 0.001 for the log-rank test; Fig. 2). A restricted cubic spline curve shows the risk of CVD mortality decreased linearly with increasing sauna sessions from one to seven (*P* value for non-linearity = 0.932) (Fig. 3). In the analyses adjusted only for age and sex, compared to participants who had one sauna session per week, the HRs of CVD mortality were 0.71 (95% CI 0.52 to 0.98) and 0.30 (0.14 to 0.64) for participants with two to three and four to seven sauna sessions per week, respectively (Table 2). Additional adjustment for several established risk factors and potential confounders minimally attenuated the HRs: 0.77 (95% CI 0.56 to 1.07) for two to three sauna bathing sessions per week and 0.36 (0.17 to 0.77) for four to seven sauna bathing sessions per week. The corresponding HRs (for two to three and four to seven sauna bathing sessions per week) remained consistent after adjustment for incident CHD as a time-varying covariate: 0.75 (95% CI 0.52 to 1.08) and 0.23 (0.08 to 0.65), respectively (Table 2). The results remained similar to additional adjustment for the temperature of sauna bathing. In the analyses by gender, there was no statistically significant evidence of associations in women, which could be attributed largely to the low event rates in the sauna exposure categories (Table 2). A test of interaction showed that the association between sauna bathing frequency and CVD mortality was not significantly modified by gender (*P* for interaction = 0.524).

**Table 2:** Hazard ratios of cardiovascular mortality according to the frequency of sauna bathing, overall and among men and women

Frequency of sauna bathing (sessions/week)	Events/total	Model 1		Model 2		Model 3		Model 4*	
		HR (95% CI)	*P* value	HR (95% CI)	*P* value	HR (95% CI)	*P* value	HR (95% CI)	*P* value
Overall									
Once	63/455	Ref		Ref		Ref		Ref	
2–3	110/1028	0.71 (0.52 to 0.98)	0.035	0.78 (0.57 to 1.08)	0.133	0.77 (0.56 to 1.07)	0.121	0.75 (0.52 to 1.08)	0.120
4–7	8/205	0.30 (0.14 to 0.64)	0.002	0.36 (0.17 to 0.76)	0.007	0.36 (0.17 to 0.77)	0.008	0.23 (0.08 to 0.65)	0.005
Men									
Once	39/177	Ref		Ref		Ref		Ref	
2–3	71/512	0.61 (0.41 to 0.90)	0.013	0.70 (0.47 to 1.03)	0.073	0.69 (0.46 to 1.03)	0.069	0.68 (0.43 to 1.09)	0.111
4–7	8/132	0.33 (0.15 to 0.71)	0.005	0.39 (0.18 to 0.84)	0.016	0.39 (0.18 to 0.84)	0.016	0.26 (0.09 to 0.75)	0.013
Women									
Once	24/278	Ref		Ref		Ref		Ref	
2–3	39/516	0.95 (0.57 to 1.57)	0.830	1.03 (0.59 to 1.77)	0.929	1.00 (0.57 to 1.74)	0.997	0.88 (0.48 to 1.60)	0.676
4–7	0/73	NE		NE		NE		NE	

Model 1: adjusted for age and gender

Model 2: model 1 plus body mass index, smoking, systolic blood pressure, serum low-density lipoprotein cholesterol, alcohol consumption, previous myocardial infarction, and type 2 diabetes

Model 3: model 2 plus physical activity (duration per week) and socio-economic status

Model 4: model 3 plus incident coronary heart disease as a time-dependent covariate

*CI* confidence interval, *HR* hazard ratio, *NE* not estimated because of zero event rate; analysis is based on 1688 participants and 181 cardiovascular deaths

*The model was limited to the population at risk and did not include those who already had coronary heart disease

**Fig. 4:**
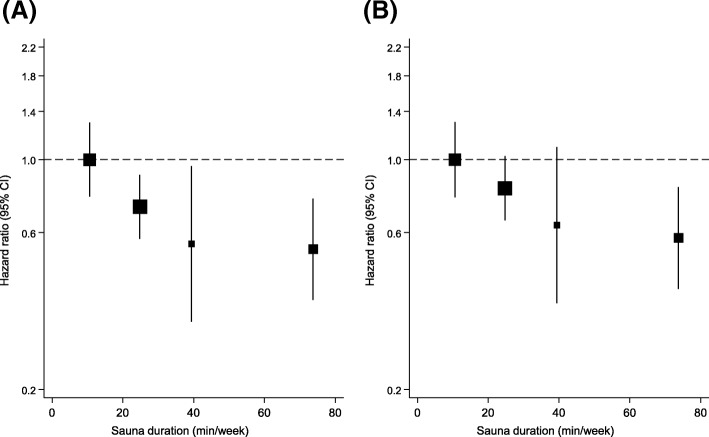
Hazard ratios for cardiovascular mortality by quartiles of the duration of sauna bathing. **a** Adjusted for age and gender. **b** Adjusted for age, gender, body mass index, smoking, systolic blood pressure, serum low-density lipoprotein cholesterol, alcohol consumption, previous myocardial infarction, and type 2 diabetes. CI, confidence interval

Cardiovascular mortality rates per 1000 person-years of follow-up across the three groups of sauna bathing duration (≤ 15, 16–45, > 45 min/week) were 9.6 (95% CI 7.5 to 12.3), 7.6 (6.2 to 9.3), and 5.1 (3.4 to 7.7), respectively. Cumulative hazard curves demonstrated a greater risk of CVD mortality among participants having a sauna bath of ≤ 15 min/week compared with the other groups (*P* = 0.028 for the log-rank test; Fig. 2). In the analysis adjusted for (i) age and sex and (ii) BMI, smoking, SBP, serum LDL-C, alcohol consumption, previous myocardial infarction, and type 2 diabetes, an inverse association was found between duration of sauna bathing and CVD mortality risk, which was potentially consistent with either a curvilinear or linear shape (Fig. 4). However, statistical tests suggested a fit with a non-linear shape (*P* for non-linearity = 0.005). After adjustment for age and gender, HR was 0.49 (0.30–0.80) for CVD mortality among participants in the highest weekly duration (> 45 min/week) compared with the lowest weekly duration (≤ 15 min/week) of sauna bathing (Table 3). The respective HR was 0.57 (0.35–0.94) after adjustment for several established CVD risk factors, and potential confounders. The respective HRs remained consistent on further adjustment for the temperature of sauna bathing. In gender-specific analyses, there was no statistically significant evidence of associations in both men and women, which could be attributed to the low event rates (Table 3). A test of interaction showed that the association between the duration of sauna bathing and CVD mortality was not significantly modified by gender (*P* for interaction = 0.314).

**Table 3:** Hazard ratios of cardiovascular mortality according to the weekly duration of sauna bathing, overall and among men and women

Duration of sauna bathing (minutes/week)	Events/total	Model 1		Model 2		Model 3		Model 4*	
		HR (95% CI)	*P* value	HR (95% CI)	*P* value	HR (95% CI)	*P* value	HR (95% CI)	*P* value
Overall									
≤ 15	62/463	Ref		Ref		Ref		Ref	
16–45	96/906	0.69 (0.50 to 0.96)	0.027	0.77 (0.55 to 1.06)	0.112	0.77 (0.55 to 1.07)	0.123	0.74 (0.51 to 1.09)	0.132
> 45	23/319	0.49 (0.30 to 0.80)	0.004	0.57 (0.35 to 0.93)	0.025	0.57 (0.35 to 0.94)	0.028	0.60 (0.34 to 1.05)	0.074
Men									
≤ 15	29/157	Ref		Ref		Ref		Ref	
16–45	72/461	0.83 (0.54 to 1.28)	0.406	0.92 (0.59 to 1.43)	0.711	0.92 (0.59 to 1.43)	0.705	1.05 (0.61 to 1.83)	0.846
> 45	17/203	0.50 (0.27 to 0.91)	0.023	0.57 (0.31 to 1.04)	0.068	0.57 (0.31 to 1.05)	0.073	0.68 (0.33 to 1.43)	0.311
Women									
≤ 15	33/306	Ref		Ref		Ref		Ref	
16–45	24/445	0.53 (0.31 to 0.89)	0.017	0.62 (0.36 to 1.08)	0.093	0.61 (0.35 to 1.07)	0.084	0.51 (0.28 to 0.91)	0.024
> 45	6/116	0.58 (0.24 to 1.39)	0.222	0.75 (0.31 to 1.83)	0.528	0.75 (0.31 to 1.85)	0.532	0.66 (0.25 to 1.76)	0.408

Model 1: adjusted for age and gender

Model 2: model 1 plus body mass index, smoking, systolic blood pressure, serum low-density lipoprotein cholesterol, alcohol consumption, previous myocardial infarction, and type 2 diabetes

Model 3: model 2 plus physical activity (duration per week) and socio-economic status

Model 4: model 3 plus incident coronary heart disease as a time-dependent covariate

*CI* confidence interval, *HR* hazard ratio; analysis is based on 1688 participants and 181 cardiovascular deaths

*The model was limited to the population at risk and did not include those who already had coronary heart disease

The associations of both frequency and duration of sauna bathing with CVD mortality risk remained consistent in the analyses that excluded the first 5 years of follow-up (Additional file 2: Tables S1–S2). In a subsidiary analysis which compared four to seven sauna sessions per week with zero to one sauna session per week, the associations were similar (Additional file 2: Table S3).

### Associations in subgroups

**Fig. 5:**
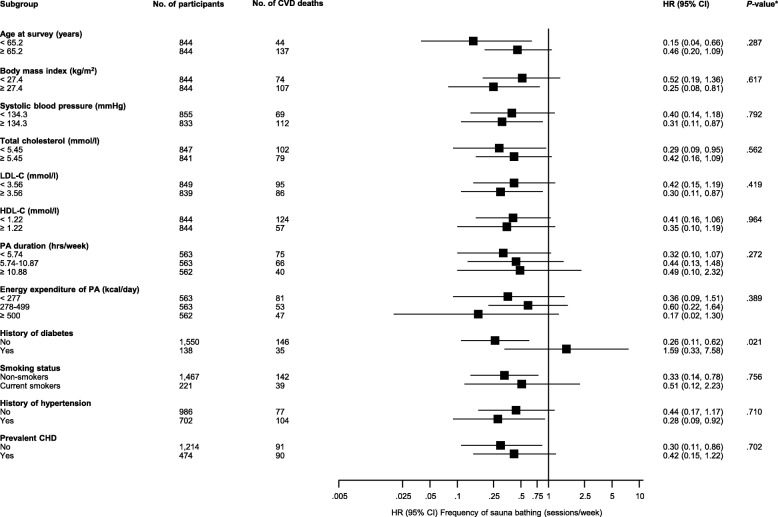
Association of the frequency of sauna bathing with cardiovascular mortality in clinically relevant subgroups. CHD, coronary heart disease; CI, confidence interval; HDL-C, high-density lipoprotein cholesterol; HR, hazard ratio; LDL-C, low-density lipoprotein cholesterol; PA, physical activity. HRs are adjusted for age, gender, body mass index, smoking, systolic blood pressure, serum low-density lipoprotein cholesterol, alcohol consumption, previous myocardial infarction, and type 2 diabetes; hazard ratios are reported comparing four to seven sauna sessions per week with one sauna session per week. * *P*-value for meta-regression

**Fig. 6:**
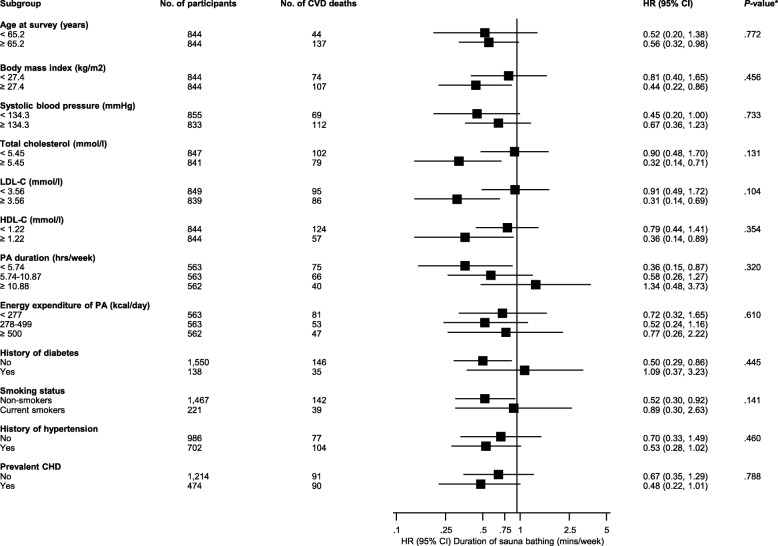
Association of the duration of sauna bathing with cardiovascular mortality in clinically relevant subgroups. CHD, coronary heart disease; CI, confidence interval; HDL-C, high-density lipoprotein cholesterol; HR, hazard ratio; LDL-C, low-density lipoprotein cholesterol; PA, physical activity. HRs are adjusted for age, gender, body mass index, smoking, systolic blood pressure, serum low-density lipoprotein cholesterol, alcohol consumption, previous myocardial infarction, and type 2 diabetes. Hazard ratios are reported comparing > 45 min of sauna bathing per week with ≤ 15 min of sauna bathing per week. * *P*-value for meta-regression

Figures 5 and 6 show the associations of frequency and duration of sauna bathing with the risk of CVD death in clinically relevant subgroups. Except for the evidence of effect modification by diabetes status for the association between sauna frequency and CVD mortality (*P* for interaction = 0.021), the associations did not vary significantly by levels or categories of several clinically relevant characteristics.

### Frequency of sauna bathing and CVD mortality risk prediction

A risk prediction model for CVD mortality containing conventional risk factors yielded a C-index of 0.7716 (95% CI 0.7382–0.8049; *P* < 0.001). After addition of information on the frequency of sauna bathing, the C-index was 0.7807 (0.7486–0.8128; *P* < 0.001), representing a significant increase of 0.0091 (0.0022–0.0160; *P* = 0.010). In addition, when investigating differences in the -2 log likelihood of the risk score with and without the inclusion of frequency of sauna bathing, the -2 log likelihood was significantly improved on the addition of information on the frequency of sauna bathing to the model (*P* for comparison = 0.019). There was a significant improvement in the classification of participants into predicted 10-year CVD mortality risk categories (NRI: 4.14%, 1.30–6.97%; *P* = 0.004). The IDI was 0.0037 (0.0002–0.0072; *P* = 0.041).

## Discussion

The findings of this long-term prospective study of over 14 years follow-up suggest that the cardiovascular benefits of sauna bathing may exist in both men and women. Our new results show that addition of information on the frequency of sauna bathing improved the prediction and reclassification of the long-term risk for CVD mortality. A higher frequency of sauna bathing sessions per week was related to a decreased risk of fatal CVD events independent of conventional cardiovascular risk factors as well as several other potential confounders. The risk of fatal CVD events decreased with increasing sauna sessions in a dose-response manner with no threshold effect. For the duration of sauna bathing per week, we observed a decrease in risk of CVD mortality with increasing duration of sauna bathing, though further work may be required to ascertain whether a curvilinear or linear shape best describes the relationship. The association was strong and also independent of several established and emerging risk factors. Except for the evidence of effect modification by diabetes status for the association between sauna frequency and CVD mortality, the associations were not modified significantly by levels or categories of several clinically relevant characteristics including gender. However, findings from the subgroup analyses should be interpreted with caution given the multiple statistical tests of interaction and the low event rates in these subgroups.

Several mechanisms can be postulated to underpin the protective effects of sauna bathing on cardiovascular mortality. Dry and hot sauna baths have been shown to increase the demands of cardiovascular function [5, 22, 38]. Sauna bathing causes an increase in heart rate which is a reaction to the body heat load. Heart rate may be elevated up to 120–150 beats per minute during sauna bathing, corresponding to low- to moderate-intensity physical exercise training for the circulatory system without active muscle work [30, 39–41]. Acute sauna exposure has been shown to produce blood pressure lowering effects [42], decrease peripheral vascular resistance [42, 43] and arterial stiffness [17, 44], and improve arterial compliance [18]. Short-term sauna exposure also activates the sympathetic nervous and the renin-angiotensin-aldosterone systems and the hypothalamus-pituitary-adrenal hormonal axis, and short-term increases in levels of their associated hormones have been reported [45]. Repeated sauna exposure improves endothelial function, suggesting a beneficial role of thermal therapy on vascular function [16–18, 46]. Long-term sauna bathing habit may be beneficial in the reduction of high systemic blood pressure [42], which is in line with previous evidence showing that blood pressure may be lower among those who are living in warm conditions with higher ambient temperature [47, 48]. We have demonstrated that regular sauna bathing is associated with a lowered risk of future hypertension [6]. Typical hot and dry Finnish sauna increases body temperature which causes more efficient skin blood flow, leading to a higher cardiac output, whereas blood flow to internal organs decreases [22]. Sweat is typically secreted at a rate which corresponds to an average total secretion of 0.5 kg during a sauna bathing session [5, 39]. Increased sweating is accompanied by a reduction in blood pressure and higher heart rate, while cardiac stroke volume is largely maintained, although a part of blood volume is diverted from the internal organs to body peripheral parts with decreasing venous return which is not facilitated by active skeletal muscle work [49]. However, it has been proposed that muscle blood flow may increase to at least some extent in response to heat stress, although sauna therapy-induced myocardial metabolic adaptations are largely unexplored [30, 50]. There is also evidence that regular long-term sauna bathing (average of two sessions per week) increases left ventricular ejection fraction [46]. Heat therapy may improve left ventricular function with decreased cardiac pre- and afterload, thereby maintaining appropriate stroke volume despite large reductions in ventricular filling pressures [16, 38, 51–53]. Additionally, previous studies have demonstrated a positive alteration of the autonomic nervous system and reduced levels of natriuretic peptides, oxidative stress, inflammation, and norepinephrine due to regular sauna therapy [15, 30, 43, 53, 54].

Our current results highlight a substantial risk reduction of fatal CVD events in men and women, with frequent sauna use of over four times per week and duration of sauna bathing of more than 45 min/week. The data suggests that a history of more frequent sauna use is associated with a decrease in the risk of fatal CVD in a linear dose-response manner. Our data was based on the total weekly duration of sauna sessions, and therefore, we are unable to make any comments regarding the minimum duration of a single session that may confer benefits. However, based on historical data, a typical sauna session usually ranges from 5 to 20 min [30], although longer sauna bathing sessions may be used depending on the individual [22]. The findings also show that frequency of sauna bathing has incremental predictive value to CVD mortality beyond conventional risk factors and has the ability to reclassify subjects across clinically relevant risk thresholds. There was no statistically significant evidence of effect modification by gender. Regular Finnish sauna bathing is safe and may have several additional health benefits. Patients with a previous myocardial infarction, stable angina pectoris, or heart failure can usually enjoy sauna bathing without any significant adverse cardiovascular effects [5, 22, 55]. In this long-term follow-up study, CVD mortality rate among most active sauna users (i.e., those participants with sauna of four to seven times per week) was 2.7 cases per 1000 person-years, indicating low risk. However, in a specific group of older individuals who are prone to orthostatic hypotension, sauna baths should be taken cautiously due to possible sudden drop in blood pressures which may occur just after a hot and dry bath [22, 30, 56]. Hypotension during and immediately after sauna can be easily prevented by appropriate fluid intake to avoid dehydration [16, 30]. Further investigation into the value of regular sauna bathing in CVD risk reduction and prevention in general populations is warranted.

Several strengths of the current study deserve consideration. This is the first prospective evaluation of the associations of both frequency and duration of sauna bathing with the risk of cardiovascular mortality in a general population including both genders. Our cohort was well characterized with a long-term follow-up period, and there were no losses to follow-up. This representative sample of middle-aged to elderly men and women who use saunas makes it possible to generalize the observed results in Northern European populations; however, prospective studies should be conducted in populations who are not accustomed to regular sauna bathing. We adjusted for a comprehensive panel of lifestyle and biological markers and included subgroup as well as risk prediction analyses using sensitive measures such as the − 2 log likelihood. Our findings were robust to the exclusion of the first 5 years of follow-up, minimizing the possibility of reverse causation bias as the explanation for our findings. Several limitations of the current study also merit consideration. As with all observational epidemiological studies, exposure assessments based on self-administered questionnaires are prone to misclassification and recall bias. Our findings from hot Finnish sauna bathing with an average temperature of approximately 80 °C cannot be directly applied to other type of steam rooms and warm water therapy which may operate at lower temperatures than a relatively dry traditional sauna and do not allow humidity changes achieved by pouring water on the heated rocks [30]. Good ventilation is a feature of a typical sauna which makes it comfortable to stay for longer periods while sauna bathing. The relatively low event rate for cardiovascular deaths (*N* = 181) precluded detailed assessment of (i) effect modification by relevant clinical characteristics on the associations and (ii) dose-response relationships of the associations. Though we accounted for many potential confounders to ensure the validity of our associations, there is a potential for residual confounding. It is possible that underlying diagnosed or undiagnosed diseases may have an effect on sauna bathing habits, suggesting reverse causality; however, our subgroup analyses according to various clinical characteristics were consistent and the associations remained robust in several sensitivity analyses, independent of many underlying clinical conditions and exclusion of the first 5 years of follow-up. Sauna bathing habits may have changed during follow-up due to probable changes in health habits or other incident diseases of participants occurring over the long period of time; however, any changes may be minimal as sauna habits are fairly stable in the Finnish population [30]. We could not account for the longer-term duration and regularity of sauna use prior to the study entry because of the lack of data. However, it is a common way to assess usual lifestyle activities using baseline questionnaires in long-term epidemiological studies. Secondly, we were unable to assess the associations between sauna bathing and CVD mortality risk when comparing people who used sauna with people who did not use sauna at all (control group). Indeed, the majority of Finnish people are accustomed to having a sauna bath regularly at least once per week, as it is traditionally part of the Finnish culture [30, 40]. The associations were unchanged in a subsidiary analysis which employed a combination of people who did not use sauna baths and those who had a single sauna session per week as a reference comparison.

In Finland, sauna is easily accessible to the majority of the population independently of socioeconomic and educational backgrounds. Sauna bathing is an activity that has been a tradition in Finland for thousands of years, and our data shows minor differences in annual salary levels according to the sauna frequency groups (in years 1998–2001; see Table 1), suggesting that sauna ownership does not correlate with financial status in Finland. It is therefore highly unlikely that these factors may explain the observed findings on sauna and fatal CVD events in this population. Indeed, SES did not differ when comparing one vs. four to seven times per week frequency groups; SES level was the highest among those using sauna two to three times per week. Based on our cross-sectional baseline data, the most frequent sauna use was directly related to the level of physical activity, BMI, energy intake, and alcohol consumption. Though there is a possibility that factors such as physical activity could potentially explain these finding, it is unlikely as our analysis accounted for the role of physical activity. Furthermore, our recent research evidence suggests that a combination of regular physical fitness and sauna baths is associated with a substantial reduction in the risk of fatal cardiovascular and all-cause mortality events compared with each modality alone [57, 58]. We have shown that even participants with low fitness levels have a reduced risk of mortality when combined with frequent (3–7 sessions per week) or infrequent (≤ 2 sessions per week) sauna use. However, mortality risk is substantially reduced in those with very high fitness levels combined with frequent use of sauna. Other studies have also reported similar findings. Iwase and colleagues demonstrated enhance metabolism in participants when isotonic exercise was performed during sauna exposure [59]. On the effects of sauna bathing on athletes, Ridge and Pyke demonstrated an augmentation in acute physiological responses when sauna exposure followed exercise [60]. In another study in which six male distance runners completed 3 weeks of post-training sauna bathing, study participants experienced an enhancement in endurance running performance [61]. The overall findings show that physical activity or fitness and sauna bathing each have independent effects on vascular disease [57, 58], which suggests that the beneficial effects of sauna bathing on CVD mortality is not due to physical activity or exercise.

## Conclusions

The current prospective study provides novel evidence that higher frequency and duration of sauna bathing may be related to a lower risk of CVD mortality in a representative population-based sample of female and male participants. In addition, the frequency of sauna bathing significantly improves the prediction and classification of the 10-year risk for CVD mortality beyond established cardiovascular risk factors. Our results extend previous evidence that sauna bathing may have cardiovascular benefits; however, further studies are still needed to confirm our findings in different populations and also assess the associations of sauna bathing habits with cause-specific cardiovascular events.

## Additional files

Additional file 1: Appendix STROBE 2007 Statement—checklist of items that should be included in reports of cohort studies. (DOCX 42 kb)

Additional file 2: **Table S1.** Hazard ratios of cardiovascular mortality according to the frequency of sauna bathing among men and women. **Table S2.** Hazard ratios of cardiovascular mortality according to the duration of sauna bathing among men and women. **Table S3.** Hazard ratios of cardiovascular mortality according to the frequency of sauna bathing among men and women, based on sauna frequency categories of 0–1, 2–3, and 4–7 times per week. (DOCX 19 kb)

## Abbreviations

95% CI: 95% confidence interval
BMI: Body mass index
CHD: Coronary heart disease
CVD: Cardiovascular disease
HDL-C: High-density lipoprotein cholesterol
HR: Hazard ratio
IDI: Integrated discrimination improvement
IQR: Interquartile range
LDL-C: Low-density lipoprotein cholesterol
NRI: Net reclassification improvement
SBP: Systolic blood pressure
SD: Standard deviation

## References

[1] Perasalo J (1988). Traditional use of the sauna for hygiene and health in Finland. Ann Clin Res.

[2] Valtakari P (1988). The sauna and bathing in different countries. Ann Clin Res.

[3] Hussain J, Cohen M (2018). Clinical effects of regular dry sauna bathing: a systematic review. Evid Based Complement Alternat Med.

[4] Leicht AS, Halliday A, Sinclair WH, D’Auria S, Buchheit M, Kenny GP, Stanley J (2018). Heart rate variability responses to acute and repeated postexercise sauna in trained cyclists. Appl Physiol Nutr Metab.

[5] Hannuksela ML, Ellahham S (2001). Benefits and risks of sauna bathing. Am J Med.

[6] Zaccardi F, Laukkanen T, Willeit P, Kunutsor SK, Kauhanen J, Laukkanen JA (2017). Sauna bathing and incident hypertension: a prospective cohort study. Am J Hypertens.

[7] Kunutsor SK, Khan H, Zaccardi F, Laukkanen T, Willeit P, Laukkanen JA (2018). Sauna bathing reduces the risk of stroke in Finnish men and women: a prospective cohort study. Neurology.

[8] Laukkanen T, Kunutsor S, Kauhanen J, Laukkanen JA (2017). Sauna bathing is inversely associated with dementia and Alzheimer’s disease in middle-aged Finnish men. Age Ageing.

[9] Laitinen LA, Lindqvist A, Heino M (1988). Lungs and ventilation in sauna. Ann Clin Res.

[10] Cox NJ, Oostendorp GM, Folgering HT, van Herwaarden CL (1989). Sauna to transiently improve pulmonary function in patients with obstructive lung disease. Arch Phys Med Rehabil.

[11] Kunutsor SK, Laukkanen T, Laukkanen JA (2017). Sauna bathing reduces the risk of respiratory diseases: a long-term prospective cohort study. Eur J Epidemiol.

[12] Nurmikko T, Hietaharju A (1992). Effect of exposure to sauna heat on neuropathic and rheumatoid pain. Pain.

[13] Isomaki H (1988). The sauna and rheumatic diseases. Ann Clin Res.

[14] Kanji G, Weatherall M, Peter R, Purdie G, Page R (2015). Efficacy of regular sauna bathing for chronic tension-type headache: a randomized controlled study. J Altern Complement Med.

[15] Brunt VE, Howard MJ, Francisco MA, Ely BR, Minson CT (2016). Passive heat therapy improves endothelial function, arterial stiffness and blood pressure in sedentary humans. J Physiol.

[16] Imamura M, Biro S, Kihara T, Yoshifuku S, Takasaki K, Otsuji Y, Minagoe S, Toyama Y, Tei C (2001). Repeated thermal therapy improves impaired vascular endothelial function in patients with coronary risk factors. J Am Coll Cardiol.

[17] Laukkanen T, Kunutsor SK, Zaccardi F, Lee E, Willeit P, Khan H, Laukkanen JA (2018). Acute effects of sauna bathing on cardiovascular function. J Hum Hypertens.

[18] Lee E, Laukkanen T, Kunutsor SK, Khan H, Willeit P, Zaccardi F, Laukkanen JA (2018). Sauna exposure leads to improved arterial compliance: findings from a non-randomised experimental study. Eur J Prev Cardiol.

[19] Laukkanen T, Khan H, Zaccardi F, Laukkanen JA (2015). Association between sauna bathing and fatal cardiovascular and all-cause mortality events. JAMA Intern Med.

[20] Salonen JT (1988). Is there a continuing need for longitudinal epidemiologic research? The Kuopio Ischaemic Heart Disease Risk Factor Study. Ann Clin Res.

[21] von Elm E, Altman DG, Egger M, Pocock SJ, Gotzsche PC, Vandenbroucke JP (2008). The Strengthening the Reporting of Observational Studies in Epidemiology (STROBE) statement: guidelines for reporting observational studies. J Clin Epidemiol.

[22] Kukkonen-Harjula K, Kauppinen K (2006). Health effects and risks of sauna bathing. Int J Circumpolar Health.

[23] Lynch JW, Kaplan GA, Cohen RD, Kauhanen J, Wilson TW, Smith NL, Salonen JT (1994). Childhood and adult socioeconomic status as predictors of mortality in Finland. Lancet.

[24] Laukkanen JA, Laaksonen D, Lakka TA, Savonen K, Rauramaa R, Makikallio T, Kurl S (2009). Determinants of cardiorespiratory fitness in men aged 42 to 60 years with and without cardiovascular disease. Am J Cardiol.

[25] Kunutsor SK, Khan H, Laukkanen JA (2015). Serum albumin concentration and incident type 2 diabetes risk: new findings from a population-based cohort study. Diabetologia.

[26] Lakka TA, Salonen JT (1992). Intra-person variability of various physical activity assessments in the Kuopio Ischaemic Heart Disease Risk Factor Study. Int J Epidemiol.

[27] Laukkanen JA, Pukkala E, Rauramaa R, Makikallio TH, Toriola AT, Kurl S (2010). Cardiorespiratory fitness, lifestyle factors and cancer risk and mortality in Finnish men. Eur J Cancer.

[28] Laukkanen JA, Kurl S, Salonen R, Rauramaa R, Salonen JT (2004). The predictive value of cardiorespiratory fitness for cardiovascular events in men with various risk profiles: a prospective population-based cohort study. Eur Heart J.

[29] Therneau TM, Grambsch PM (2000). Modeling survival data: extending the cox model.

[30] Laukkanen JA, Laukkanen T, Kunutsor SK (2018). Cardiovascular and other health benefits of sauna bathing: a review of evidence. Mayo Clin Proc.

[31] Groenwold RH, Klungel OH, Grobbee DE, Hoes AW (2011). Selection of confounding variables should not be based on observed associations with exposure. Eur J Epidemiol.

[32] Harrell FE, Lee KL, Mark DB (1996). Multivariable prognostic models: issues in developing models, evaluating assumptions and adequacy, and measuring and reducing errors. Stat Med.

[33] Pencina MJ, D’Agostino RB, D’Agostino RB, Vasan RS (2008). Evaluating the added predictive ability of a new marker: from area under the ROC curve to reclassification and beyond. Stat Med.

[34] Pencina MJ, D’Agostino RB, Steyerberg EW (2011). Extensions of net reclassification improvement calculations to measure usefulness of new biomarkers. Stat Med.

[35] Gupta S, Rohatgi A, Ayers CR, Willis BL, Haskell WL, Khera A, Drazner MH, de Lemos JA, Berry JD (2011). Cardiorespiratory fitness and classification of risk of cardiovascular disease mortality. Circulation.

[36] Cook NR (2007). Use and misuse of the receiver operating characteristic curve in risk prediction. Circulation.

[37] Harrell FEJ (2001). Regression modeling strategies.

[38] Crandall CG, González-Alonso J (2010). Cardiovascular function in the heat-stressed human. Acta Physiol (Oxf).

[39] Vuori I (1988). Sauna bather’s circulation. Ann Clin Res.

[40] Kukkonen-Harjula K, Oja P, Laustiola K, Vuori I, Jolkkonen J, Siitonen S, Vapaatalo H (1989). Haemodynamic and hormonal responses to heat exposure in a Finnish sauna bath. Eur J Appl Physiol Occup Physiol.

[41] Leppäluoto J, Tuominen M, Väänänen A, Karpakka J, Vuori J (1986). Some cardiovascular and metabolic effects of repeated sauna bathing. Acta Physiol Scand.

[42] Gayda M, Paillard F, Sosner P, Juneau M, Garzon M, Gonzalez M, Belanger M, Nigam A (2012). Effects of sauna alone and postexercise sauna baths on blood pressure and hemodynamic variables in patients with untreated hypertension. J Clin Hypertens (Greenwich).

[43] Radtke T, Poerschke D, Wilhelm M, Trachsel LD, Tschanz H, Matter F, Jauslin D, Saner H, Schmid JP (2016). Acute effects of Finnish sauna and cold-water immersion on haemodynamic variables and autonomic nervous system activity in patients with heart failure. Eur J Prev Cardiol.

[44] Heinonen I, Laukkanen JA (2018). Effects of heat and cold on health, with special reference to Finnish sauna bathing. Am J Physiol Regul Integr Comp Physiol.

[45] Kukkonen-Harjula K, Kauppinen K (1988). How the sauna affects the endocrine system. Ann Clin Res.

[46] Ohori T, Nozawa T, Ihori H, Shida T, Sobajima M, Matsuki A, Yasumura S, Inoue H (2012). Effect of repeated sauna treatment on exercise tolerance and endothelial function in patients with chronic heart failure. Am J Cardiol.

[47] Lewington S, Li L, Sherliker P, Guo Y, Millwood I, Bian Z, Whitlock G, Yang L, Collins R, Chen J (2012). Seasonal variation in blood pressure and its relationship with outdoor temperature in 10 diverse regions of China: the China Kadoorie Biobank. J Hypertens.

[48] Kunutsor SK, Powles JW (2010). The effect of ambient temperature on blood pressure in a rural West African adult population: a cross-sectional study. Cardiovasc J Afr.

[49] Crandall CG, Wilson TE, Marving J, Vogelsang TW, Kjaer A, Hesse B, Secher NH (2008). Effects of passive heating on central blood volume and ventricular dimensions in humans. J Physiol.

[50] Heinonen I, Brothers RM, Kemppainen J, Knuuti J, Kalliokoski KK, Crandall CG (2011). Local heating, but not indirect whole body heating, increases human skeletal muscle blood flow. J Appl Physiol (1985).

[51] Fujita S, Ikeda Y, Miyata M, Shinsato T, Kubozono T, Kuwahata S, Hamada N, Miyauchi T, Yamaguchi T, Torii H (2011). Effect of Waon therapy on oxidative stress in chronic heart failure. Circ J.

[52] Tei C, Horikiri Y, Park JC, Jeong JW, Chang KS, Toyama Y, Tanaka N (1995). Acute hemodynamic improvement by thermal vasodilation in congestive heart failure. Circulation.

[53] Miyata M, Kihara T, Kubozono T, Ikeda Y, Shinsato T, Izumi T, Matsuzaki M, Yamaguchi T, Kasanuki H, Daida H (2008). Beneficial effects of Waon therapy on patients with chronic heart failure: results of a prospective multicenter study. J Cardiol.

[54] Sutkowy P, Woźniak A, Boraczyński T, Mila-Kierzenkowska C, Boraczyński M (2014). The effect of a single Finnish sauna bath after aerobic exercise on the oxidative status in healthy men. Scand J Clin Lab Invest.

[55] Kauppinen K (1997). Facts and fables about sauna. Ann N Y Acad Sci.

[56] Keast ML, Adamo KB (2000). The Finnish sauna bath and its use in patients with cardiovascular disease. J Cardpulm Rehabil.

[57] Kunutsor SK, Khan H, Laukkanen T, Laukkanen JA (2018). Joint associations of sauna bathing and cardiorespiratory fitness on cardiovascular and all-cause mortality risk: a long-term prospective cohort study. Ann Med.

[58] Laukkanen JA, Laukkanen T, Khan H, Babar M, Kunutsor SK (2018). Combined effect of sauna bathing and cardiorespiratory fitness on the risk of sudden cardiac deaths in Caucasian men: a long-term prospective cohort study. Prog Cardiovasc Dis.

[59] Iwase S, Kawahara Y, Nishimura N, Nishimura R, Sugenoya J, Miwa C, Takada M (2014). Effects of isotonic and isometric exercises with mist sauna bathing on cardiovascular, thermoregulatory, and metabolic functions. Int J Biometeorol.

[60] Ridge BR, Pyke FS (1986). Physiological responses to combinations of exercise and sauna. Aust J Sci Med Sport.

[61] Scoon GS, Hopkins WG, Mayhew S, Cotter JD (2007). Effect of post-exercise sauna bathing on the endurance performance of competitive male runners. J Sci Med Sport.

