# Supplementary material for: Sauna bathing is associated with reduced cardiovascular mortality and improves risk prediction in men and women: a prospective cohort study
Source: BMC Med. 2018 Nov 29;16:219. doi: 10.1186/s12916-018-1198-0 (PMC6262976; doi:10.1186/s12916-018-1198-0)
Supplement: Supplementary file 2 — Table S1. Hazard ratios of cardiovascular mortality according to the frequency of sauna bathing among men and women. Table S2. Hazard ratios of cardiovascular mortality according to the duration of sauna bathing among men and women. Table S3. Hazard ratios of cardiovascular mortality according to the frequency of sauna bathing among men and women, based on sauna frequency categories of 0–1, 2–3, and 4–7 times per week. (DOCX 19 kb) [file 12916_2018_1198_MOESM2_ESM.docx]

**Table S1.** Hazard ratios of cardiovascular mortality according to the frequency of sauna bathing among men and women

| **First five years of follow-up excluded** | | |
| --- | --- | --- |
| **Frequency of sauna bathing (number of participants)** | HR (95% CI) | *P-*value |
| Once/week (437) | reference |  |
| 2-3 times/week (986) | 0.70 (0.49 to 1.00) | 0.051 |
| 4-7 times/week (199) | 0.37 (0.17 to 0.83) | 0.016 |
| *P*-value for trend |  | 0.006 |

CI, confidence interval; HR, hazard ratio

Hazards ratios are adjusted for age, gender, body mass index, smoking, systolic blood pressure, serum low-density lipoprotein cholesterol, alcohol consumption, previous myocardial infarction, type 2 diabetes, physical activity (duration per week), and socio-economic status

**Table S2.** Hazard ratios of cardiovascular mortality according to the duration of sauna bathing among men and women

| **First five years of follow-up excluded** | | |
| --- | --- | --- |
| **Duration of sauna bathing (number of participants)** | HR (95% CI) | *P-*value |
| ≤ 15 min/week (446) | reference |  |
| 16-45 min/week (870) | 0.76 (0.53 to 1.10) | 0.148 |
| > 45 min/week (306) | 0.47 (0.26 to 0.84) | 0.011 |
| *P*-value for trend |  | 0.015 |

CI, confidence interval; HR, hazard ratio

Hazards ratios are adjusted for age, gender, body mass index, smoking, systolic blood pressure, serum low-density lipoprotein cholesterol, alcohol consumption, previous myocardial infarction, type 2 diabetes, physical activity (duration per week), and socio-economic status

**Table S3.** Hazard ratios of cardiovascular mortality according to the frequency of sauna bathing among men and women, based on sauna frequency categories of 0-1, 2-3 and 4-7 times per week

| **Frequency of sauna bathing (times/week)** | **Events/**  **Total** | **Model 1** |  | **Model 2** |  | **Model 3** |  | **Model 4*** |  |
| --- | --- | --- | --- | --- | --- | --- | --- | --- | --- |
|  |  | HR (95% CI) | *P-*value | HR (95% CI) | *P-*value | HR (95% CI) | *P-*value | HR (95% CI) | *P-*value |
| 0-1 | 66 / 499 | ref |  | ref |  | ref |  | ref |  |
| 2-3 | 110 / 1,028 | 0.73 (0.54 to 0.99) | 0.046 | 0.79 (0.58 to 1.08) | 0.141 | 0.78 (0.57 to 1.07) | 0.129 | 0.75 (0.52 to 1.07) | 0.111 |
| 4-7 | 8 / 205 | 0.31 (0.15 to 0.66) | 0.002 | 0.36 (0.17 to 0.77) | 0.008 | 0.37 (0.17 to 0.78) | 0.009 | 0.23 (0.08 to 0.65) | 0.006 |
| *P*-value for trend |  |  | 0.001 |  | 0.006 |  | 0.007 |  | 0.004 |

CI, confidence interval; HR, hazard ratio; analysis is based on 1,732 participants and 184 cardiovascular deaths

Model 1: Adjusted for age and gender

Model 2: Model 1 plus body mass index, smoking, systolic blood pressure, serum low-density lipoprotein cholesterol, alcohol consumption, previous myocardial infarction and type 2 diabetes

Model 3: Model 2 plus physical activity (duration per week), and socio-economic status

Model 4: Model 3 plus incident coronary heart disease as a time-dependent covariate

*the model was limited to the population at risk and did not include those who already had coronary heart disease
